# Effects of Oxygen on Lattice Defects in Single-Crystalline Mg_2_Si Thermoelectrics

**DOI:** 10.3390/nano13071222

**Published:** 2023-03-30

**Authors:** Kei Hayashi, Sota Kawamura, Yusuke Hashimoto, Noboru Akao, Zhicheng Huang, Wataru Saito, Kaichi Tasaki, Koichi Hayashi, Tomohiro Matsushita, Yuzuru Miyazaki

**Affiliations:** 1 Department of Applied Physics, Graduate School of Engineering, Tohoku University, Sendai 980-8579, Japan; 2 Graduate School of Science and Technology, Nara Institute of Science and Technology, Ikoma 630-0192, Japan; 3 Department of Materials Science, Graduate School of Engineering, Tohoku University, Sendai 980-8579, Japan; 4 Department of Physical Science and Engineering, Nagoya Institute of Technology, Nagoya 466-8555, Japan; 5 Japan Synchrotron Radiation Research Institute (JASRI), Sayo 679-5198, Japan

**Keywords:** Mg_2_Si single crystals, lattice defects, electronic structure calculations, photoelectron holography

## Abstract

Lattice defect engineering has attracted attention due to its ability to develop thermoelectric materials with low thermal conductivity. For Mg_2_Si single crystals (SCs), Si vacancy (V_Si_) defects can be introduced and consequently result in the formation of dislocation cores. These lattice defects confer Mg_2_Si SCs with a lower thermal conductivity compared to Mg_2_Si polycrystals. To reveal a mechanism for the stabilisation of V_Si_ in the Mg_2_Si SCs, we investigated the effects of oxygen (O) on lattice defects by performing electronic structure calculations, secondary ion mass spectrometry, X-ray photoelectron spectroscopy, and photoelectron holography. On the basis of these calculations, we predicted that O stabilised the formation of V_Si_ when it was located at the Si site or at an interstitial site. All experiments confirmed the presence of O inside the Mg_2_Si SCs. However, O was suggested to be located not at the specific site in the crystal lattice of Mg_2_Si but at dislocation cores. The interaction between O and the dislocation cores in the Mg_2_Si SC is expected to immobilise dislocation cores, leading to the stabilisation of V_Si_ formation.

## 1. Introduction

Lattice defect engineering is key to developing energy-harvesting materials such as photovoltaic, dielectric, and thermoelectric (TE) materials [1,2,3]. Lattice defects such as point defects and dislocation cores should be reduced in photovoltaic and dielectric materials to achieve high energy-conversion efficiency. Although the introduction of lattice defects into TE materials also affects TE properties (the Seebeck coefficient, electrical conductivity, and thermal conductivity) [4,5,6,7,8,9,10,11,12,13,14,15,16,17,18,19,20,21,22,23,24,25,26,27,28,29,30,31], it can improve TE performance mainly because lattice thermal conductivity (*κ*_L_) decreases due to enhanced phonon scattering by lattice defects [5,11,16,19,23,24,29,30,31]. Thus, investigating which point defect is formed and how it works in TE materials is important.

In this study, we focused on Mg_2_Si, which has attracted considerable attention as a potential TE material [3,8,21,32,33]. Mg_2_Si has an antifluorite type structure (space group: *Fm*$\bar{3}$*m*), wherein the 8*c*(1/4 1/4 1/4) and the 4*a*(0 0 0) sites were occupied by Mg and Si, respectively. The most probable point of the defect in Mg_2_Si was theoretically predicted to be an interstitial defect in which the 4*b*(1/2 1/2 1/2) site was partially occupied by Mg [34,35,36]. This interstitial Mg (Mg_i_) was present in synthesised Mg_2_Si-based polycrystals (PCs) [6,7,21,37]. However, recent studies have revealed that undoped and boron (B)-doped Mg_2_Si single crystals (SCs) contain Si vacancy (V_Si_) defects [17]. In addition, V_Si_ defects induce edge dislocation cores in these Mg_2_Si-based SCs [27]. As a result of the presence of V_Si_ defects and dislocation cores, Mg_2_Si-based SCs exhibited lower *κ*_L_ than Mg_2_Si PCs [27]. The difference in point defects between Mg_2_Si-based PCs and SCs may arise from preparation temperature, which is typically 1123 K for Mg_2_Si-based PCs [6,7] and 1413 K for Mg_2_Si-based SCs [17,27]. The higher preparation temperature for Mg_2_Si-based SCs can induce V_Si_. However, Mg_2_Si with V_Si_ is not expected to be stable, because V_Si_ has higher formation energy than Mg_i_ [34,35,36]. One possibility for the stabilisation of V_Si_ in Mg_2_Si is its incorporation with oxygen (O). A theoretical study predicted that O intercalation into the 4*b*(1/2 1/2 1/2) site resulted in p-type Mg_2_Si [38]. This prediction was consistent with the findings of an experimental study on postannealed Mg_2_Si thin films [39], which also suggested that Mg vacancy (V_Mg_) defects were formed through the postannealing process. The other possibility for interaction between dislocation cores and O. In a Si SC, O diffuses during preparation and precipitates at dislocation cores [40,41,42,43]. Subsequently, location of Si and O atoms were rearranged around dislocation cores, making the dislocation cores stable [41,42,43]. A similar O precipitation at the dislocation cores may occur in Mg_2_Si-based SCs, leading to the stabilization of V_Si_.

Motivated by the above studies, we investigated the effects of O on V_Si_ and the dislocation cores in Mg_2_Si SCs. By using first-principles electronic structure calculations, we examined whether the incorporation of O stabilised the formation of V_Si_. Furthermore, we performed secondary ion mass spectrometry (SIMS), X-ray photoelectron spectroscopy (XPS), and photoelectron holography to verify the existence of O in the prepared Mg_2_Si SCs. In particular, on the basis of photoelectron holograms, we discussed the three-dimensional atomic arrangements of Mg, Si, and O.

## 2. Calculation and Experimental Methods

We used the full-potential linearised augmented plane wave (FLAPW) method implemented in the Wien2k code [44] and the Korringa-Kohn-Rostoker (KKR) method under the coherent potential approximation implemented in the AkaiKKR code [45] to reduce the cost for the electronic structure calculation. Although the GW approximation, using the Green’s function *G* and the screened Coulomb potential *W*, has been reported to be the most accurate method for the calculation of Mg_2_Si, Mg_2_Ge, and Mg_2_Sn [46], the FLAPW and/or KKR methods are known to be sufficient for determining the conduction type and comparing the total energy, *E*, of different crystal structure models [8,10,12,18,19,20,22,25,28]. The local exchange-correlation potential in generalised gradient approximation was used for calculation through the FLAPW method. Twelve crystal structure models with a 2 × 2 × 2 cubic supercell were used, as shown in Figures S1–S3. Model 1 is ‘Mg_64_Si_32′_, which contains 64 Mg atoms at the 8*c* site and 32 Si atoms at the 4*a* site without any point defects. Model 2a is ‘Mg_64_Si_32_+Mg_i_’, which contains one additional Mg_i_. Models 2b and 2c contain one Mg_i_ and an O atom at the 4*b* site (O_i_) with different distances between Mg_i_ and O_i_, namely, ‘Mg_64_Si_32_+Mg_i_+O_i_’. Model 3a is designated as ‘Mg_63_Si_32′_, i.e., one V_Mg_ exists in a supercell. Models 3b and 3c also contain one V_Mg_ and one O_i_ with different distances between V_Mg_ and O_i_ and are expressed as ‘Mg_63_Si_32_+O_i_’. One V_Si_ exists in Model 4a, and one additional O_i_ exists in Models 4b and 4c; these models are represented as ‘Mg_64_Si_31′_ and ‘Mg_64_Si_31_+O_i_’, respectively. Models 3d and 4d are denoted as ‘Mg_63_Si_32_+O_Mg_’ and ‘Mg_64_Si_31_+O_Si_’, respectively, where an O atom can be substituted for an Mg/Si atom. We investigated the stability of the point defects in each crystal structure model on the basis of the calculation of total energy *E*. For the calculation, the *E* values of Mg, Si, and/or O atoms were added to equalise the number of atoms.

(1) $$E=E\left({\mathrm{Mg}}_{64-a}{\mathrm{Si}}_{32-b}+c{\mathrm{Mg}}_{i}+dO_{i}+eO_{\mathrm{Mg}}+fO_{\mathrm{Si}}\right)+\left(1+a-c\right)E\left(\mathrm{Mg}\right)+b\cdot E\left(\mathrm{Si}\right)+\left(1-d-e-f\right)E\left(O\right),$$

where *a*–*f* is a constant (0 or 1). The numbers of *k*-points in the Brillouin zone for each model, Mg, and Si, were 108, 2028, and 3430, respectively. We also calculated the electronic density of states (DOS) to examine the conduction type. In the KKR method, the generalised gradient approximation and Perdew–Burke–Ernzerhof functional were used. The angular momentum cut-off was 2. The imaginary part added to the Fermi energy was set to 0.0001 Ry to calculate the DOS of Mg_2_Si, Mg_2_Si_0.97_, Mg_2_Si_0_._97_+0.03O_Si_, Mg_2_Si_0_._99_, and Mg_2_Si_0_._99_+0.01O_Si_. The number of *k*-points was 2168.

The preparation procedure of the Mg_2_Si SC that contained V_Si_ and dislocation cores is described elsewhere [17]. The depth profiles of O, carbon (C), hydrogen (H), Si, and MgSi in the prepared Mg_2_Si SCs were determined through SIMS (IONTOF, TOF-SIMS5-100) in negative polarity mode. The XPS spectra of the Mg_2_Si SC were acquired in a vacuum (1.2 × 10^−5^ Pa) by using Al Kα radiation as a light source (ThermoFisher Scientific, Theta Probe). A cleavage surface was obtained just before the sample was introduced into the XPS chamber. Surface etching was performed in the chamber by using Ar ion milling. Photoelectron holography, a type of atomic resolution holography that can directly reveal a three-dimensional local structure around a target atom [47,48,49,50,51,52,53,54,55,56,57,58,59,60,61,62,63], was performed by using soft X-ray as a light source at the beamline 25SU [64] of the synchrotron radiation facility Super Photon ring-8 GeV (SPring-8), Japan. A wide-angle display-type retarding field analyser was used for the measurement [65]. A cleaved surface was obtained after the introduction of the sample into the vacuum chamber. The vacuum pressure during the measurement was 6.5 × 10^−8^ Pa. From photoelectron holography, an atomic arrangement of O on W(110) [62] and a defect structure including O at the interface between Al_2_O_3_ and diamond [63] was revealed. Thus, the position of O in the Mg_2_Si SC can be determined if a structure around O has a long-range order.

## 3. Results and Discussion

**Figure 1:**
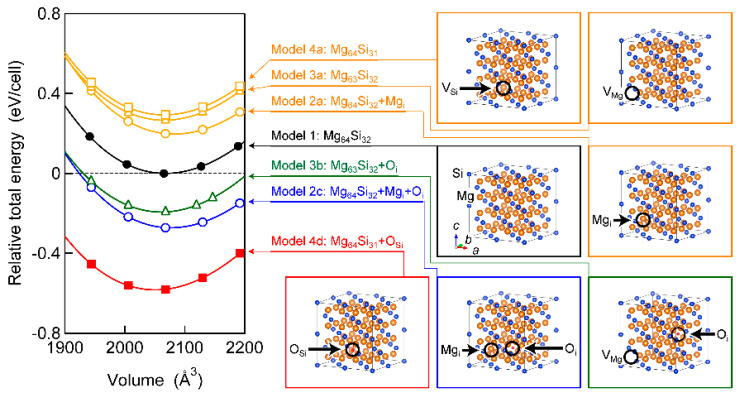
Volume dependence of the total energy of seven crystal structure models relative to the minimum energy of Model 1. Crystal structures were drawn using VESTA [66].

By using the FLAPW method, we investigated whether point defects in each supercell were stable or not. Figures S1–S3 show that the *E* of Models 2a (Mg_64_Si_32_+Mg_i_), 3a (Mg_63_Si_32_), and 4a (Mg_64_Si_31_) were higher than that of Model 1 (Mg_64_Si_32_). This result indicates that the formation energy of Mg_i_, V_Mg_, and V_Si_ was positive (+0.197 eV/cell, +0.266 eV/cell, and +0.293 eV/cell, respectively). Consistent with the results of previous studies [34,35,36], V_Si_ showed the highest *E*, i.e., it had the highest formation energy, among the point defects. However, Models 2b, 2c, 3b, 3c, 3d, 4b, 4c, and 4d, which all contained O, exhibited a lower *E* than Model 1, indicating that the introduction of O could stabilise the formation of Mg_i_, V_Mg_, and V_Si_. Models 2c (Mg_64_Si_32_+Mg_i_+O_i_), 3b (Mg_63_Si_32_+O_i_), and 4d (Mg_64_Si_31_+O_Si_) had the lowest *E* in the cases of Mg_i_, V_Mg_, and V_Si_, respectively. The *E* values of Models 1, 2a, 3a, 4a, 2c, 3b, and 4d are summarised in Figure 1. Although Model 4a had the highest *E*, the introduction of O into the Si site (Model 4d) resulted in the lowest *E* value. The formation energy of Mg_i_+O_i_ (Model 2c), V_Mg_+O_i_ (Model 3b), and O_Si_ (Model 4d) were −0.272 eV/cell, −0.193 eV/cell, and −0.583 eV/cell, respectively. In addition, the *E* values of Models 4b and 4c, which contained V_Si_ and O_i_ (see Figure S3), respectively, were lower or equal to those of Models 2c and 3b, which contained Mg_i_/V_Mg_ and O_i_, respectively. The formation energy of V_Si_+O_i_ (Models 4b and 4c) was −0.211 eV/cell and −0.296 eV/cell, respectively. In other words, the incorporation of O stabilised the formation of V_Si_ rather than Mg_i_ and V_Mg_, regardless of whether O was located at the Si site or at the interstitial site. (The formation energy of each defect is summarised in Table S1).

**Figure 2:**
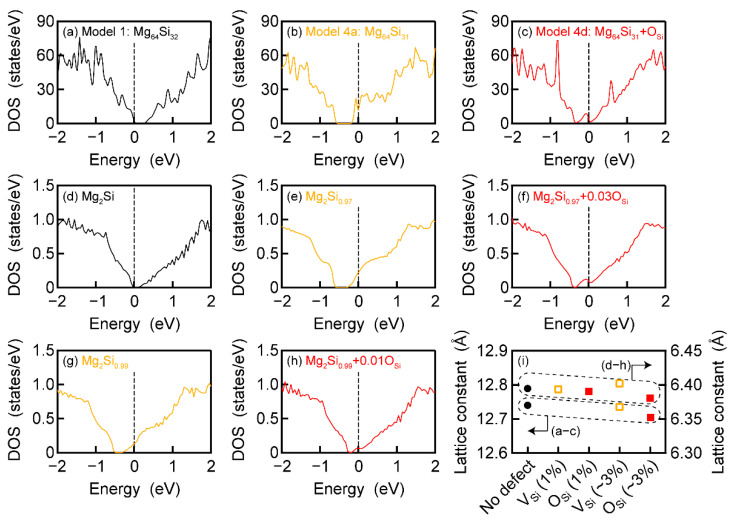
Electronic density of states (DOS) for (**a**) Model 1, (**b**) Model 4a, and (**c**) Model 4d shown in Figure 1. Electronic DOS of the 1 × 1 × 1 cubic cell of (**d**) Mg_2_Si, (**e**) Mg_2_Si_0_._97_, (**f**) Mg_2_Si_0_._97_+0.03O_Si_, (**g**) Mg_2_Si_0_._99_, and (**h**) Mg_2_Si_0_._99_+0.01O_Si_. (**i**) Lattice constant used for the calculation of (**a**–**h**), which provided the minimum total energy.

Next, we examined the conduction type of Models 1, 4a, and 4d by calculating their DOS through the FLAPW method. By introducing one V_Si_ defect into Mg_64_Si_32_, p-type conductivity changed to n-type one (see Figure 2a,b). The band gap increased with the introduction of the V_Si_ defect from 0.11 eV to 0.19 eV. Similar to the introduction of one Mg_i_ into Mg_64_Si_32_ and Mg_216_Si_108_ [21], the substitution of one O for the Si site caused an in-gap state just at the bottom of the conduction band (Figure 2c). Given that the Fermi level (*E*_F_) was located inside the conduction band, Mg_64_Si_31_+O_Si_ was found to have n-type conductivity. These calculation results were reproduced using the KKR method, as shown in Figure 2d–f. Note that the fraction of V_Si_ or O_Si_ in the crystal structure models used for the calculation of Figure 2b,c,e,f was approximately 3%. By contrast, the V_Si_ fraction in the prepared Mg_2_Si SCs was reported to be approximately 1%. Thus, we calculated the DOS of Mg_2_Si_0_._99_ by using the KKR method (Figure 2g). The n-type conductivity was confirmed, which was consistent with a previous calculation and used another KKR code [67]. The increase in the band gap was also found in the KKR calculation. The band gap of Mg_2_Si was 0.10 eV, whereas that of Mg_2_Si_0_._99_ and Mg_2_Si_0_._97_ was 0.15 eV and 0.20 eV, respectively. The band gap of Mg_2_Si and Mg_2_Si_0_._97_ was in good agreement with the FLAPW calculation. For Mg_2_Si_0_._99_+0.01O_Si_ (Figure 2h), the in-gap state became smaller than that shown in Figure 2c,f, but *E*_F_ continued to exist inside the conduction band. From the above calculations, we found that the presence of V_Si_ or O_Si_ led to n-type conductivity of Mg_2_Si. This result was consistent with the experiments showing that the prepared Mg_2_Si SCs had a negative Seebeck coefficient, i.e., they had n-type conductivity [17]. Figure 2i presents the lattice constant at the minimum *E*, which was used for the calculation of the results given in Figure 2a,h. The introduction of V_Si_ or O_Si_ reduced the lattice constant. Experimentally, the smaller lattice constant resulted in an increase in the V_Si_ fraction in Mg_2_Si SCs [17]. A similar tendency was reported for the prepared Mg_2_Sn SCs, wherein the V_Mg_ fraction increased with the decrease in the lattice constant [16,27]. As expected from the above calculations, O can be O_i_ or O_Si_ to stabilise V_Si_ if it exists in the crystal lattice of Mg_2_Si.

**Figure 3:**
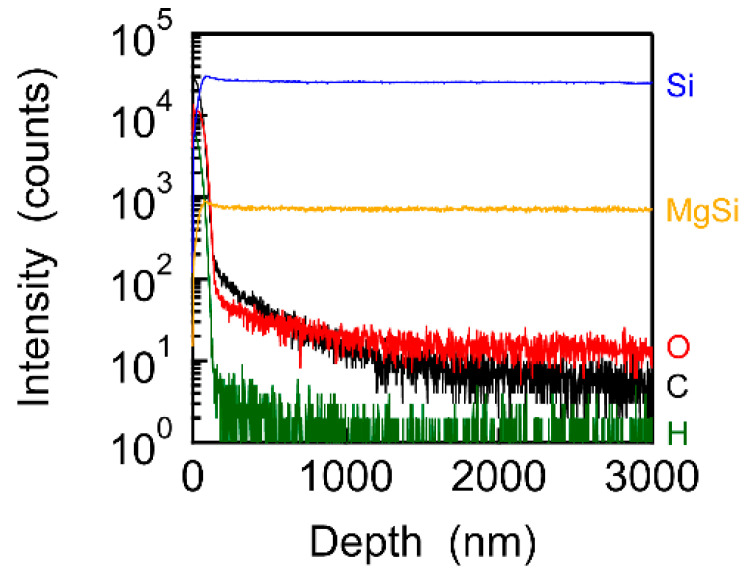
Depth profiles of O, C, H, Si, and MgSi of the Mg_2_Si SC.

We acquired the depth profiles of O, C, H, Si, and MgSi by using SIMS, as shown in Figure 3, to investigate whether O was present or absent in the Mg_2_Si SC. O, C, and H were mainly detected below 200 nm, indicating that the surface of the Mg_2_Si SC was contaminated with O, C, and H. As the depth increased to 200 nm, the H intensity decreased to a noise level (<10 counts), and Si and MgSi were clearly detected instead. The C intensity also decreased and reached the noise level at 1600 nm. These results suggested that surface contamination with C and H could be eliminated by etching or cleaving the Mg_2_Si SC. On the other hand, O intensity decreased but remained constant above 1200 nm. Thus, O was expected to exist in the Mg_2_Si SC after etching or cleaving.

**Figure 4:**
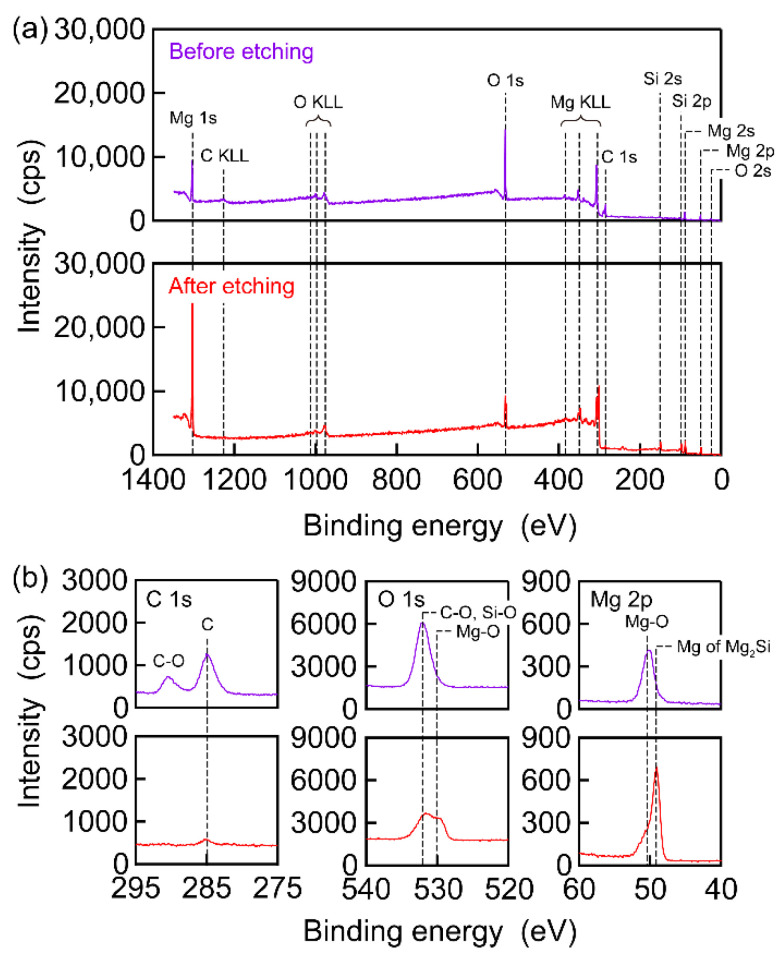
(**a**) X-ray photoelectron spectroscopy survey spectra and (**b**) core level spectra of the Mg_2_Si SC before and after etching (upper and lower, respectively).

The presence of O inside the Mg_2_Si SC was also confirmed by acquiring the XPS spectra before and after etching, as shown in Figure 4a. C and O peaks, in addition to Mg and Si peaks, were observed before etching. This finding indicated that the surface of the Mg_2_Si SC was contaminated and had oxidised. In fact, the surface of Mg_2_Si SC is known to oxidise when it is placed in the atmosphere [68]. After etching, the C peak disappeared, but the O peaks remained. Additional detailed information is provided in Figure 4b. Before etching, two peaks were observed in the C 1s XPS spectrum (upper-left figure). These peaks, which were assigned to C-O (290 eV [69]) and C (285 eV), were diminished after etching (lower-left figure). A broad peak consisting of C-O (532 eV), Si-O (532 eV [70]), and Mg-O (530 eV) components were observed in the O 1s XPS spectrum before etching (upper-middle figure). The surface oxidation and/or the presence of O inside the Mg_2_Si SC were ascribed to the Si-O and Mg-O components. By etching the surface, the broad O 1s peak changed into double peaks (lower-middle figure) due to a decrease in C-related contamination. In other words, the C-O component at 532 eV disappeared, making the Si-O and Mg-O components more evident after etching. The remaining Si-O and Mg-O components indicated that O existed inside the Mg_2_Si SC. The removal of surface oxidation was confirmed by comparing the Mg 2p XPS spectra before and after etching (upper-right and lower-right figures, respectively). A broad peak was observed in the spectra. This peak could be separated into two components at 50 eV and 49 eV. Such components were also found in an Sb-doped Mg_2_Si_0_._4_Sn_0_._6_ PC and were assigned to the Mg of Mg_2_Si (50 eV) and Mg_i_ (49 eV) components [71]. However, this assignment can be disputed, given the absence of Mg_i_ in the Mg_2_Si SC [17]. Studies [72,73,74] have reported that the Mg-O and Mg peaks of Mg_2_Si components appeared in the Mg 2p XPS spectra at lower and higher binding energies, respectively. In consideration of these studies, as well as the O 1s XPS spectra in this study, we concluded that the components observed at 50 eV and 49 eV were Mg-O and Mg in Mg_2_Si components, respectively. Etching the surface changed the intensity ratio of these components. After etching, the Mg-O component decreased, whereas the Mg of the Mg_2_Si component increased. This result indicates that although the oxidised surface had been removed, O remained in the Mg_2_Si SC.

**Figure 5:**
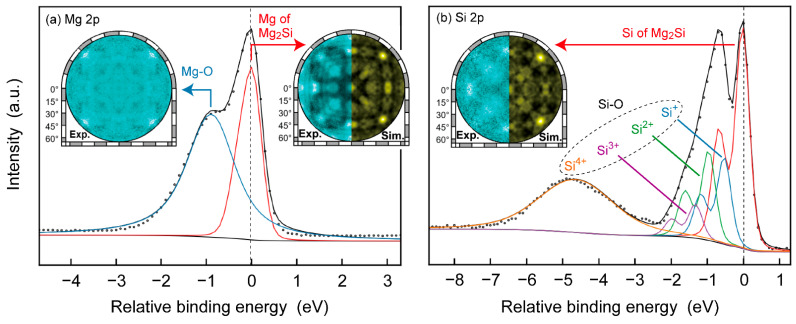
(**a**) Mg 2p and (**b**) Si 2p spectra taken in photoelectron holography measurements of the Mg_2_Si SC (dots). Each spectrum is deconvoluted into several components (coloured curves). The sum of all components is drawn as a black curve. Insets are holograms derived from measured Mg, Mg-O, and Si components (blue-black colour scale) and simulated holograms (yellow-black colour scale).

We further proved that O was present in Mg_2_Si SC through the use of photoelectron holography. Figure 5a shows the Mg 2p spectrum (dots), which could be deconvoluted into two components (blue and red curves). The spin-orbit splitting for each component was set at 0.6 eV. In consideration of the XPS results shown previously, we assigned the components at higher and lower binding energies to Mg-O and Mg in Mg_2_Si, respectively. The difference in the energy position between the two components was 1 eV and was consistent with that in the Mg 2p XPS spectra. The insets in Figure 5a are the holograms derived from each component (blue-black colour scale). In particular, the hologram for the component at a lower binding energy well coincided with the hologram simulated using the regular atomic arrangement of the Mg and Si atoms of Mg_2_Si around an emitter Mg atom (yellow-black colour scale). Thus, the assignment of the components in the Mg 2p spectrum was found to be valid. The Si 2p spectrum (dots) is shown in Figure 5b. The spin-orbit splitting for each component was set at 0.6 eV. Similar to a previous result [68], a component of Si for Mg_2_Si at the lowest binding energy and Si-O components at a higher binding energy were present in the Si 2p spectrum (coloured curves). The Si-O components were Si^+^, Si^2+^, Si^3+^, and Si^4+^ at 0.5 eV, 0.9 eV, 1.3 eV, and 4.5 eV, respectively. The energy positions of these components were evaluated relative to that of the Si component and were found to correspond reasonably to values in the literature [75,76,77]. An inset in Figure 5b is an experimental hologram derived from the Si component (left) and a simulated hologram constructed on the basis of the regular atomic arrangement of the Mg and Si atoms of Mg_2_Si around an emitter Si atom (right). Given that the experimental hologram coincided with the simulated one, the assignment of the components was confirmed to be valid for the Si 2p spectrum. Although the cleavage surface was prepared in a vacuum, the Mg-O and Si-O components were found in Mg 2p and Si 2p spectra, respectively, verifying that O was present inside the Mg_2_Si SC. The remaining O in an Ar gas and/or the aluminum crucible was used for the preparation of the Mg_2_Si SC, which could be a source of O.

**Figure 6:**
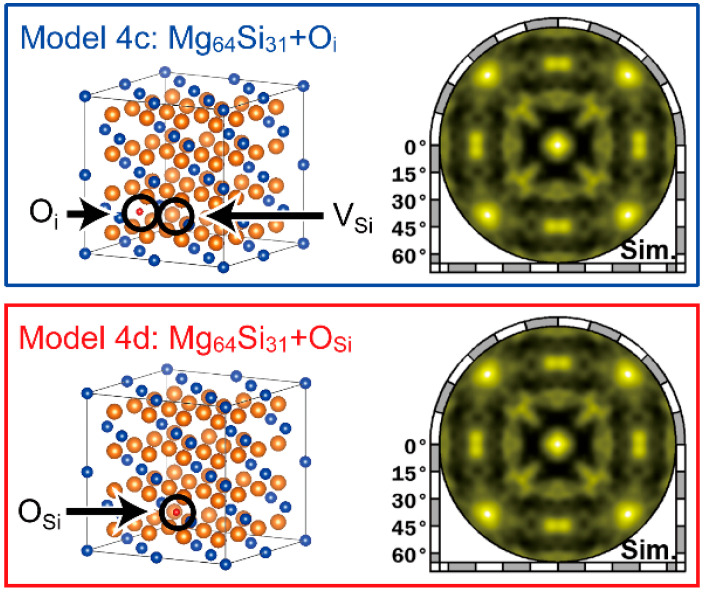
Simulated holograms of Model 4c (Mg_64_Si_31_+ O_i_) and Model 4d (Mg_64_Si_31_+O_Si_).

Here, we discuss the position of O in the Mg_2_Si SC. A hologram derived from the Mg-O component is shown in the inset of Figure 5a, which is rather unclear compared with those derived from the Mg and Si components. We reconstructed a simulated hologram of Model 4c (Mg_64_Si_31_+O_i_) and Model 4d (Mg_64_Si_31_+O_Si_), as shown in Figure 6, to examine the position. The characteristic features in the simulated holograms were not identified in the experimental hologram. Thus, we could not conclude that O existed at a specific site of the crystal lattice of Mg_2_Si. Instead, O was highly likely to be present at the dislocation cores. O was likely diffused and segregated to the dislocation cores during crystal growth. O segregation resulted in the immobilisation of the dislocation cores through the reconstruction of Mg, Si, and O atom locations around the dislocation cores. The reconstruction process reduced the total energy, which, in turn, stabilised V_Si_. Note that reconstructed atomic arrangements lacked a long-range order; therefore, the hologram derived from the Mg-O component was featureless. In the future, other experiments, such as x-ray absorption spectroscopy and positron annihilation spectroscopy, will be performed to determine the position of O precisely.

In summary, using the FLAPW and KKR methods, we theoretically predicted that the presence of O stabilised the formation of Mg_i_, V_Mg_, and V_Si_ in Mg_2_Si SCs. The formation energy of V_Si_+O_i_ (Models 4b and 4c) and O_Si_ (Model 4d) was lower or equal to that of the other lattice defects, indicating that Mg_2_Si SCs with V_Si_ were stabilised through the incorporation of O. The O_Si_ defect showed the lowest formation energy of the −0.583 eV/cell. The calculated DOS indicated that the electrical conduction of Mg_2_Si changed from p-type to n-type by introducing V_Si_. The n-type conductivity was maintained for the additional introduction of O into Mg_2_Si with V_Si_. The presence of C and O in the prepared Mg_2_Si SC was revealed by SIMS and XPS. C existed in the vicinity of the surface of the Mg_2_Si SC, whereas O was present not only at the surface but also inside the Mg_2_Si SC. The presence of O was also confirmed by photoelectron holography measurements on the cleavage surface of the Mg_2_Si SC. In the Mg 2p spectrum, Mg and Mg-O components were observed, which was consistent with the XPS measurements. In the Si 2p spectrum, Si and Si-O components were present. An experimental hologram derived from the Mg and Si components was reproduced by a simulated hologram constructed on the basis of the regular atomic arrangement of the Mg and Si atoms of Mg_2_Si around an Mg and Si atom, respectively. On the other hand, an experimental hologram derived from the Mg-O component was featureless and did not coincide with a simulated hologram of Mg_2_Si with V_Si_ or O_Si_. This result indicated that O could be located at dislocation cores, not at the interstitial site or at the Si site in the Mg_2_Si SC. The Mg_2_Si SC with V_Si_ may have become stable due to the interaction between O and the dislocation cores. The effect of O as well as the higher preparation temperature of the Mg_2_Si SC, are the reasons for the formation of V_Si_ in the Mg_2_Si SC.

## Data Availability

Not applicable.

## Supplementary Materials

The following supporting information can be downloaded at: https://www.mdpi.com/article/10.3390/nano13071222/s1, Figure S1: Volume dependence of the total energy for the four crystal structure models relative to the minimum energy of Model 1. Crystal structures are drawn by using VESTA [66]; Figure S2: Volume dependence of the total energy for the five crystal structure models relative to the minimum energy of Model 1; Figure S3: Volume dependence of the total energy for the five crystal structure models relative to the minimum energy of Model 1; Table S1: Formation energy of Mg_i_, V_Mg_, V_Si_, and their complex defects in combination with O.
