# Supplementary material for: Effects of Oxygen on Lattice Defects in Single-Crystalline Mg2Si Thermoelectrics
Source: Nanomaterials (Basel). 2023 Mar 30;13(7):1222. doi: 10.3390/nano13071222 (PMC10097258; doi:10.3390/nano13071222)
Supplement: Supplementary file 1 [file nanomaterials-13-01222-s001.zip › nanomaterials-2221850-supplementary.pdf]

Effects of oxygen on lattice defects in single-crystalline Mg_2Si thermoelectrics

Kei Hayashi [1,*], Sota Kawamura [2], Yusuke Hashimoto [2], Noboru Akao [3], Zhicheng Huang [1], Wataru Saito [1], Kaichi Tasaki [1], Koichi, Hayashi [4,5], Tomohiro Matsushita [2], and Yuzuru Miyazaki [1]

[1] Department of Applied Physics, Graduate School of Engineering, Tohoku University, Sendai 980-8579, Japan
[2] Graduate School of Science and Technology, Nara Institute of Science and Technology, Ikoma 630-0192, Japan
[3] Department of Materials Science, Graduate School of Engineering, Tohoku University, Sendai 980-8579, Japan
[4] Department of Physical Science and Engineering, Nagoya Institute of Technology, Nagoya 466-8555, Japan
[5] Japan Synchrotron Radiation Research Institute (JASRI), Sayo 679-5198, Japan
* Correspondence: kei.hayashi.b5@tohoku.ac.jp; Tel.: +81-22-795-4637

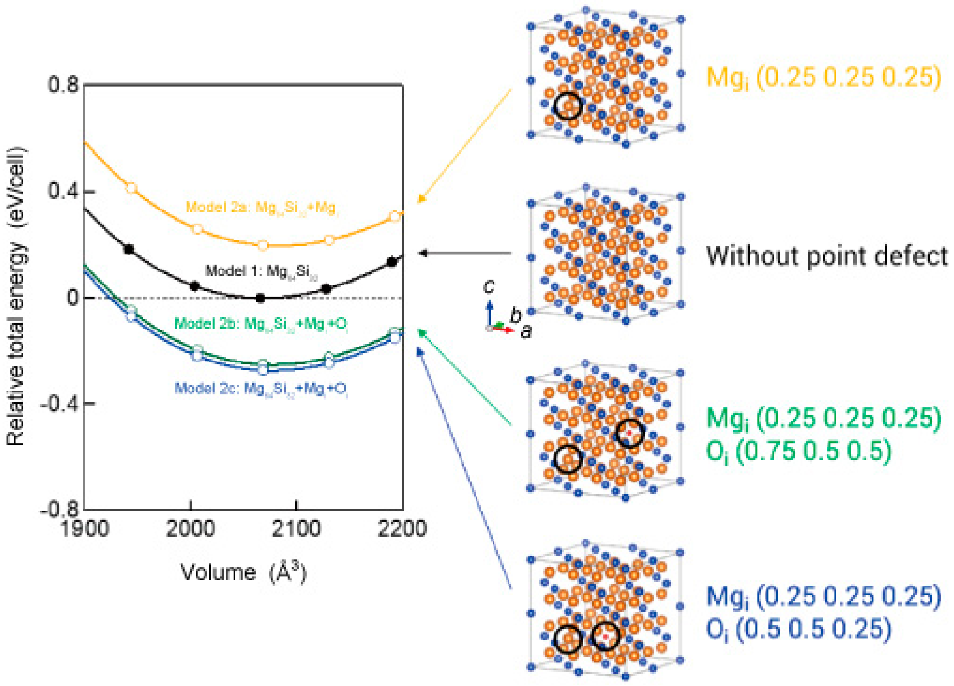

Figure S1. Volume dependence of the total energy for the four crystal structure models relative to the minimum energy of Model 1. Crystal structures are drawn by using VESTA [1].

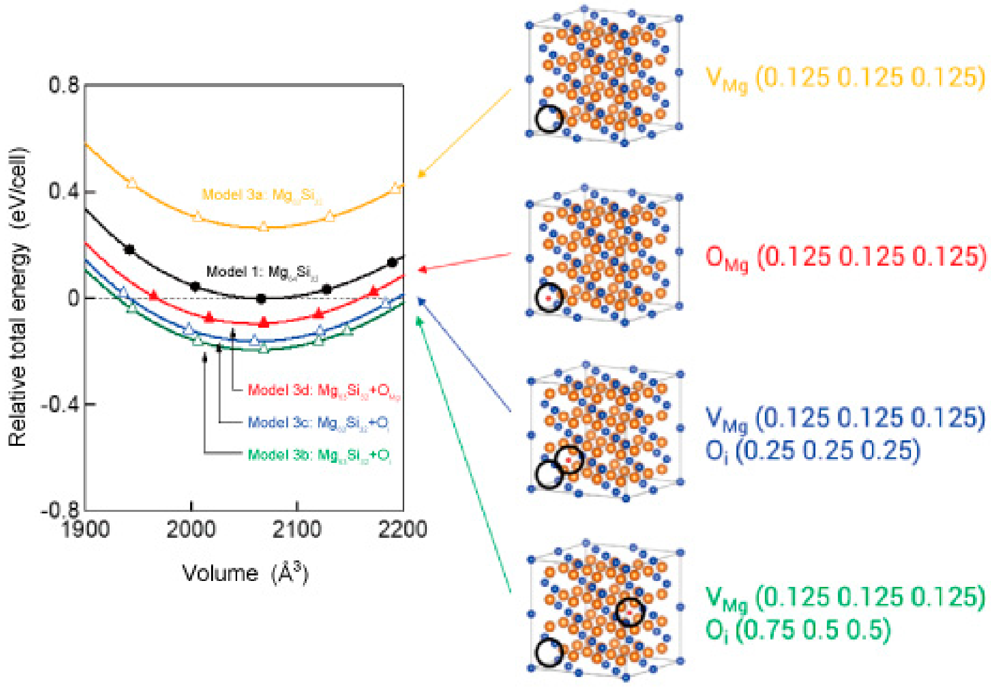

Figure S2. Volume dependence of the total energy for the five crystal structure models relative to the minimum energy of Model 1.

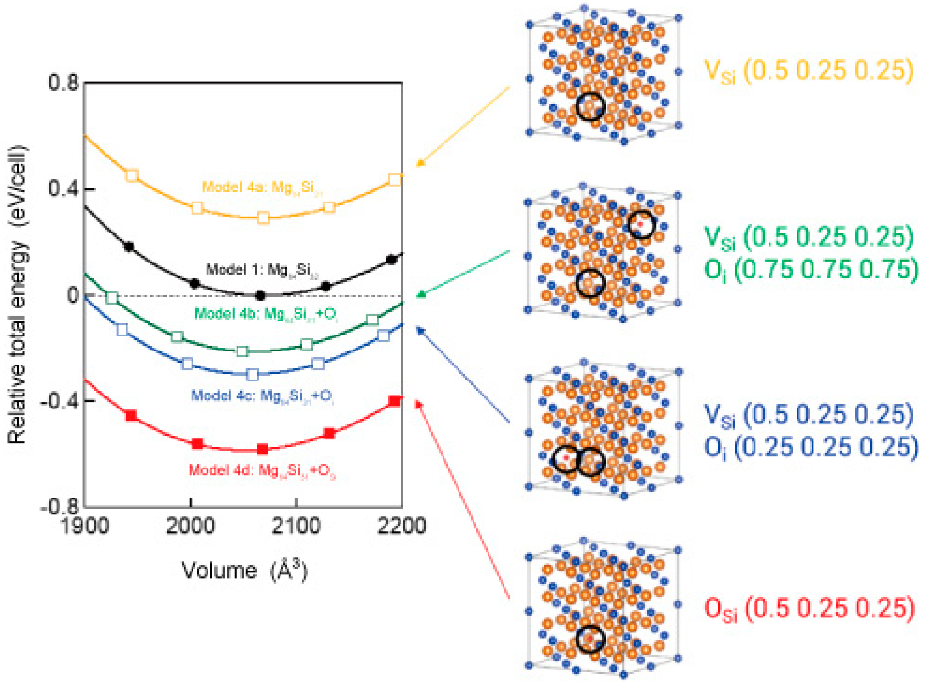

Figure S3. Volume dependence of the total energy for the five crystal structure models relative to the minimum energy of Model 1.

Table S1. Formation energy of Mg_i, V_{Mg}, V_{Si}, and their complex defects in combination with O.

Model	Defect	Formation energy (eV/cell)
2a	Mg_i	+0.197
2b	Mg_i+O_i	-0.251
2c	Mg_i+O_i	-0.272
3a	V_{Mg}	+0.266
3b	V_{Mg}+O_i	-0.193
3c	V_{Mg}+O_i	-0.161
3d	O_{Mg}	-0.094
4a	V_{Si}	+0.293
4b	V_{Si}+O_i	-0.211
4c	V_{Si}+O_i	-0.296
4d	O_{Si}	-0.583

References

1. Momma, K.; Izumi, F. VESTA 3 for three-dimensional visualization of crystal, volumetric and morphology data. *J. Appl. Crystallogr.* **2011**, *44*, 1272-1276.
